# Supplementary material for: Heat-Labile Toxin from Enterotoxigenic Escherichia coli Causes Systemic Impairment in Zebrafish Model
Source: Toxins (Basel). 2021 Jun 12;13(6):419. doi: 10.3390/toxins13060419 (PMC8231604; doi:10.3390/toxins13060419)
Supplement: Supplementary file 1 [file toxins-13-00419-s001.zip › toxins-1170504-supplementary/Supplemental Materials/toxins-1170504-Supplementary-Figure S1.pdf]

Supplementary Materials: Heat-Labile Toxin from Enterotoxigenic *Escherichia Coli* Causes Systemic Impairment in Zebrafish Model

Camila Henrique, Maria Alice Pimentel Falcão, Luciana De Araújo Pimenta, Adolfo Luís Almeida Maleski, Carla Lima, Thais Mitsunari, Sandra Coccuzzo Sampaio, Mônica Lopes-Ferreira and Roxane Maria Fontes Piazza

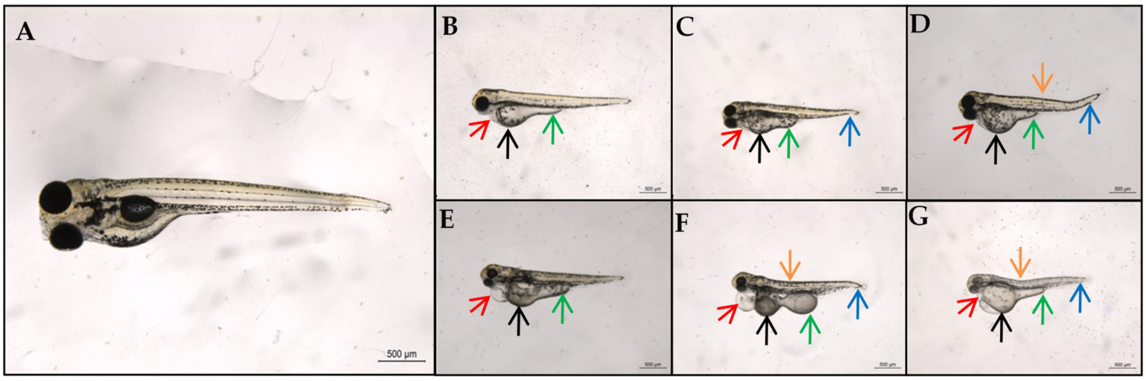

Figure S1. Phenotypes observed in zebrafish larvae, after FET test with different concentrations of LT-I toxin for 96 h. (**A**) Healthy zebrafish larva (control); (**B**–**G**) Larva evaluated after intoxication with LT-I (**B**) 0.31 nM. (**C**) 0.62 nM. (**D**) 1.5 nM. (**E**) 2.5 nM. (**F** and **G**) 5 nM. Red arrow: enlarged cardiac cavity; Black arrow: yolk edema; Green arrow: urogenital edema; Blue arrow: caudal curvature; Orange arrow: curvature of the spine.
